# Pneumatosis cystoides intestinalis with pneumoperitoneum in an 87-years-old male patient: a case report

**DOI:** 10.1093/jscr/rjac103

**Published:** 2022-03-22

**Authors:** Rodrigo Piltcher-da-Silva, Vivian Laís Sasaki, Matheus Antonio Chiconelli Zangari, Felipe Melloto Gallotti, Bruna Freitas Saenger, Mariana Piltcher-Recuero, Gabriela de Melo Rocha, Marco Raeder da Costa, Júlio Cezar Uili Coelho

**Affiliations:** Division of General and Digestive Surgery, Hospital Nossa Senhora das Graças, Curitiba, Brazil; Division of General and Digestive Surgery, Hospital Nossa Senhora das Graças, Curitiba, Brazil; Division of General and Digestive Surgery, Hospital Nossa Senhora das Graças, Curitiba, Brazil; Division of General and Digestive Surgery, Hospital Nossa Senhora das Graças, Curitiba, Brazil; Division of General and Digestive Surgery, Hospital Nossa Senhora das Graças, Curitiba, Brazil; Division of General and Digestive Surgery, Hospital Nossa Senhora das Graças, Curitiba, Brazil; Department of Radiology, Hospital Nossa Senhora das Graças, Curitiba, Brazil; Division of General and Digestive Surgery, Hospital Nossa Senhora das Graças, Curitiba, Brazil; Division of General and Digestive Surgery, Hospital Nossa Senhora das Graças, Curitiba, Brazil

## Abstract

Pneumatosis cystoides intestinalis (PCI) is a rare condition, characterized by gas-filled cysts in the intestinal wall. The mesentery and intra-abdominal ligaments can be affected. PCI is classified as primary or secondary and associated with multiple predisposing factors. An asymptomatic 87-year-old man underwent an abdominal tomography for follow-up of bladder carcinoma. The examination revealed intestinal and mesenteric pneumatosis associated with pneumoperitoneum. At laparoscopy, intestinal and mesenteric pneumatosis without intestinal infarction was identified. He was discharged on the fifth postoperative day. PCI is a benign condition that can be confused with mesenteric ischemia. Treatment is conservative, with periodic clinical evaluations. Surgical procedure is unnecessary for its diagnosis or management.

## INTRODUCTION

Pneumatosis cystoides intestinalis (PCI) is a rare pathology with a worldwide incidence of 0.03% and is three times more common in males [1–4]. Pneumoperitoneum may also occur as a consequence of subserosal bleb rupture [4, 5]. The presence of pneumatosis intestinalis associated with pneumoperitoneum strongly indicates intestinal ischemia, a life-threatening condition that should be ruled out [2, 5, 6].

The pathogenesis of the disease is still not well understood, with the following theories: bacterial, mechanical, biochemical and pulmonary origin [5–7]. PCI classically affects the digestive tract, with rare involvement of the mesentery, omentum and hepatogastric ligament [2]. This condition causes a wide spectrum of non-specific symptoms [1, 8–10]. In 3% of cases it may be associated with complications, such as volvus, perforation, hemorrhage and obstruction [1, 5]. There is still no consensus on the need of diagnostic laparoscopy [5, 11].

Regarding PCI, its development and its management is still in discussion. The radiologic presentation is similar to intestinal ischemic process, a life-threatening disease. We present a case of a patient with PCI associated with pneumoperitoneum.

### CASE REPORT

**Figure 1:**
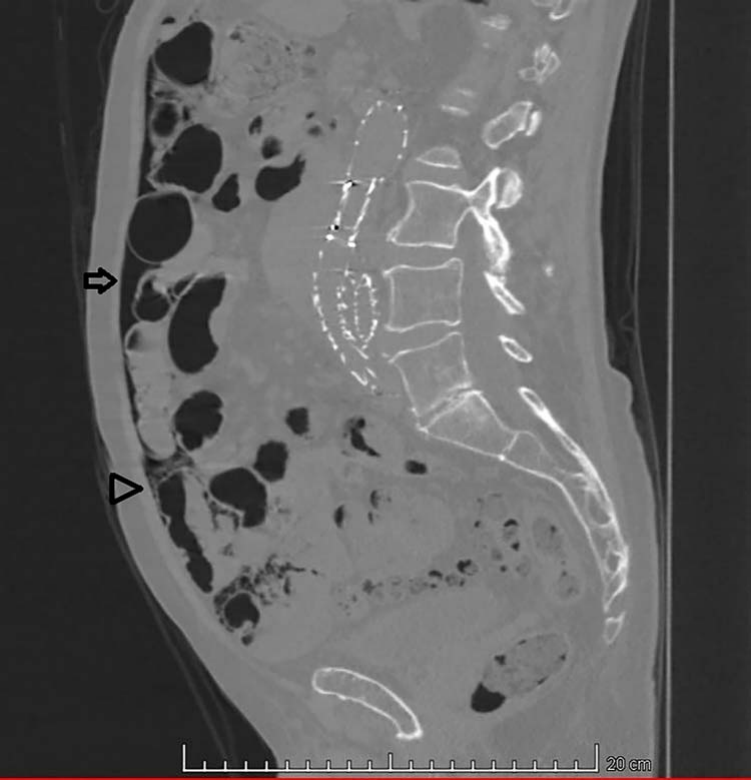
Sagittal CT image in lung window: (arrow) pneumoperitoneum and (arrow head) ‘bubbles’ within the walls of small intestine segment and the adjacent mesentery, featuring pneumatosis cystoides intestinalis and mesenteric.

An 87-year-old male patient came to the radiology department to perform an abdominal computed tomography (CT) for follow-up of a bladder urothelial carcinoma. Upon CT evaluation, diffuse intestinal pneumatosis associated with pneumoperitoneum was identified on the exam (Fig. 1). The patient was called to return promptly to the hospital.

**Figure 2:**
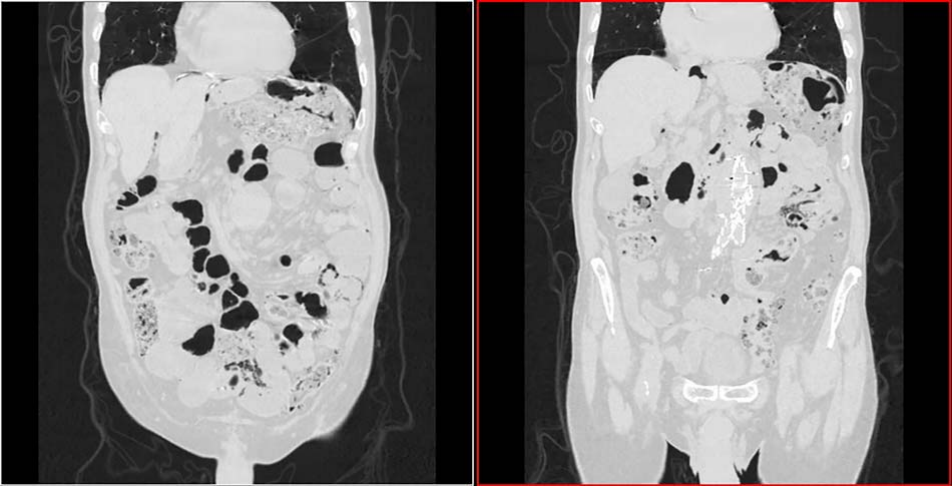
Transversal CT image in lung window showing pneumoperitoneum and pneumatosis intestinalis.

**Figure 3:**
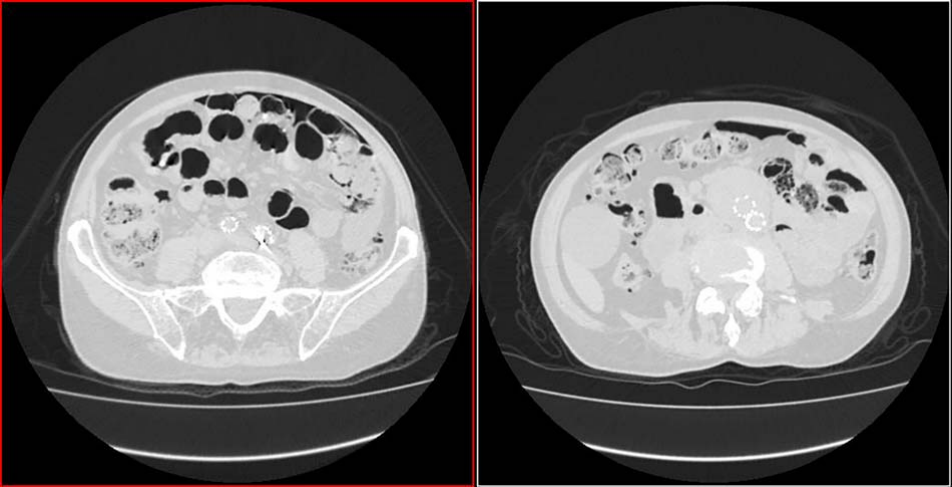
Coronal CT image in lung window showing pneumoperitoneum and ‘bubbles’ within the walls of small intestine and in the mesentery.

He arrived at the emergency department walking and with no clinical distress. He was complaining of hematuria for a month and worsening of chronic constipation in the last 2 days. Physical examination demonstrated discrete abdominal distention. Laboratory tests showed normal c-reactive protein (CRP), white blood cell count and lactate level. A new CT revealed no change in the previous findings: intestinal pneumatosis and pneumoperitoneum (Figs 2 and 3).

The general surgery service was then called. In order to exclude intestinal ischemia or other cause of gastrointestinal perforation, a diagnostic videolaparoscopy was performed. Sparse pneumatosis cystoides intestinalis of the small intestine with greater involvement of the proximal jejunum was identified. There were no signs of gastrointestinal ischemic process or perforation.

Colonoscopy showed diffuse diverticular disease of the colon, with no evidence of inflammatory process. Upper digestive endoscopy was unremarkable. He had an uneventful recovery and was discharged from the hospital on the fifth postoperative day.

## DISCUSSION

PCI can be divided into primary (15%) or secondary (85%) form, the first is idiopathic and the latter has its etiology associated with lung disease, gastrointestinal disease, abdominal trauma, connective tissue diseases, drugs or malnutrition [2, 5, 7, 9, 10]. There are four main theories for its pathogenesis. The bacterial hypothesis: presence of aerogenic bacteria inside the intestinal wall that produces gas; the mechanical hypothesis: increased intraluminal pressure with epithelial damage that allows passage of gas from the lumen to the intestinal wall; the biochemical hypothesis: increased hydrogen gas production from carbohydrate fermentation exerts pressure within the intestinal lumen and is forced through the mucosa; the pulmonary hypothesis: pulmonary disease such as chronic obstructive pulmonary disease (COPD) and interstitial pneumonia may cause alveolar rupture with the release of gas that follows the path of the vessels reaching the mesentery [2, 3, 5–7, 12, 13]. Coughing causes abrupt changes in intra-abdominal pressure and may be a contributory factor [2, 3].

The predisposing factors are chronic inflammation, trauma, scleroderma, systemic lupus erythematosus, granulomatosis with polyangiitis, amyloidosis, myeloma, dermatomyositis, COPD, connective tissue disease, Crohn’s disease, malnutrition, dysbacteriosis, gastrointestinal dysmotility, alpha-glucosidase inhibitor, corticotherapy, immune dysfunctions, bone marrow transplantation, lung transplantation, graft versus host disease and use of trichloroethylene [1–3, 5–7, 10, 14]. Thus, it is observed that the impairment of immune function and the chronic inflammation appear as main factors [2, 10].

Some predisposing factors have a clear relationship to at least one of the four main theories. Glucocorticoids can induce atrophy and fibrosis of the intestinal mucosa, in addition to altering immune function [5, 8]. Chronic constipation can promote bacterial overproliferation, increasing intraluminal hydrogen production and leaking to the intestinal wall [3]. Alpha-glucosidase inhibitors reduce carbohydrate absorption, which will be fermented by the bacterial flora, producing gas, elevate intraluminal pressure and promoting gas infiltration through the mucosal barrier [8]. Diabetes promotes peristaltic dysfunction in consequence of autonomic neuropathy, elevates intraluminal pressure and predisposes to infiltration of gas through the wall [8].

The cause of PCI in our patient could not be determined. Although he had stage III urinary bladder neoplasm he had no history of chemotherapy nor use of medications continuously. Chronic constipation that was aggravated recently might be a predisposing factor.

The colon is affected in 36–78% and small intestine in 20–51.6% of cases and both are affected in 2–22% of cases [2, 7, 11, 13]. The presentation can be chronic or acute and consists of abdominal pain, abdominal distension, nausea, vomiting, diarrhea, constipation, anorexia, weight loss and flatulence [4, 7, 11].

Although not pathognomonic, CT features is the main diagnostic of this condition, multiple cystic lesions in the intestinal wall in ‘grape bunches’ [7, 11]. These changes may be present in the mesentery, omentum and ligaments. CT can also determine the underlying etiology or identify complications [5, 6]. Leukocytosis, elevated CRP and lactate greater than two are also indications that may have some secondary cause [4, 10, 12]. Laparoscopy is often performed due to doubts about the benignity of the case, since the direct evaluation of the intestine brings safety to the diagnosis [8].

Endoscopic exams evaluate the presence of submucosal cysts and rule out the presence of associated pathologies such as lymphoma, carcinoma, inflammatory disease and polyposis [1, 13]. In the anatomopathological examination of the cyst wall, when resected, a chronic inflammatory process is usually found [3].

PCI can be self-limited and conservative treatment is effective in 90% of cases, using oxygen and antibiotics if necessary [3, 5, 12]. Although hyperbaric oxygen therapy has been used, its efficacy has not been established [4]. Laparoscopy should be considered in patients with pneumatosis intestinalis if leukocytosis and metabolic acidosis are present or if clinical worsening occurs [5–7, 11].

## CONCLUSION

PCI is a challenging and rare condition. It should be suspected in patients with pneumatosis intestinalis and pneumoperitoneum associated with mild symptoms and innocent laboratory evaluation. Diagnosis can be performed using CT. PCI recognition is important in order to avoid unnecessary surgical procedures.

## FUNDING

This study did not receive any specific grant from funding agencies in the public, commercial or non-profit sectors.

## CONFLICT OF INTEREST STATEMENT

The authors have no conflicts of interests to declare that are relevant to this article.

## ETHICS APPROVAL

This study complies with international ethical standards.
